# Delayed Anemia after Treatment with Injectable Artesunate in the Democratic Republic of the Congo: A Manageable Issue

**DOI:** 10.4269/ajtmh.14-0149

**Published:** 2014-10-01

**Authors:** Christian Burri, Giovanfrancesco Ferrari, Henry Maggi Ntuku, Antoinette Tshefu Kitoto, Stephan Duparc, Pierre Hugo, Didier Kalemwa Mitembo, Christian Lengeler

**Affiliations:** Swiss Tropical and Public Health Institute, Basel, Switzerland; University of Basel, Basel, Switzerland; Kinshasa School of Public Health, Kinshasa, Democratic Republic of the Congo; Medicines for Malaria Venture, Geneva, Switzerland

## Abstract

Cases of delayed hemolytic anemia have been described after treatment with injectable artesunate, the current World Health Organization (WHO)–recommended first-line drug for the treatment of severe malaria. A total of 350 patients (215 [61.4%] < 5 years of age and 135 [38.6%] ≥ 5 years of age) were followed-up after treatment with injectable artesunate for severe malaria in hospitals and health centers of the Democratic Republic of the Congo. Complete series of hemoglobin (Hb) measurements were available for 201 patients. A decrease in Hb levels between 2 and 5 g/dL was detected in 23 (11.4%) patients during the follow-up period. For five patients, Hb levels decreased below 5 g/dL during at least one follow-up visit. All cases of delayed anemia were clinically manageable and resolved within one month.

Acute *Plasmodium falciparum* malaria and improperly treated uncomplicated malaria can progress rapidly, especially in young children and almost invariably results in death.^1^ The second edition of the WHO guidelines for the treatment of malaria updated in April 2011 recommends injectable artesunate for the management of severe malaria in all age groups and epidemiologic settings.^2^,^3^

In early 2012, the National Malaria Control Program of the Democratic Republic of the Congo adopted the new guidelines. To assess the feasibility and acceptability of use of the new drug in the Democratic Republic of the Congo, we conducted a longitudinal operational study (Malaria Treatment with Injectable Artesunate [MATIAS]).

In mid-January 2013, during the recruitment phase of MATIAS, the Centers for Disease Control and Prevention (CDC) reported 19 cases of delayed hemolytic anemia in hyperparasitemic travelers in the second and third weeks after treatment with parenteral artesunate.^4^ The hemolysis resolved and Hb levels improved in all patients within 4–8 weeks after artesunate therapy.

In view of the large scale deployment of parenteral artesunate at national level, these results warranted further investigations. On the basis of this report, the MATIAS protocol was amended to extend the follow-up of patients from 7 to 28 days after parenteral treatment with artesunate and to include weekly measurements of Hb levels. We present the results of a sub-study conducted within the MATIAS study in eight sites in the Democratic Republic of the Congo.

A total of 350 patients who fulfilled the WHO criteria for severe *P. falciparum* malaria were treated with intravenous artesunate (Guilin Pharmaceuticals, Shanghai, China). Details of history and clinical assessment obtained by local physicians and nurses were collected for all patients. A thick blood smear was prepared and a rapid diagnostic test was conducted at admission for each patient. Thereafter, a thick blood film was prepared every 12 hours during the first 24 hours and then every 24 hours until results were negative or patient discharge. The patients were treated with injectable artesunate at a dose of 2.4 mg/kg given at admission, at 12 hours, at 24 hours, and then once a day until the patient was able to tolerate oral medication, in accordance with WHO recommendations. Parenteral treatment was completed by giving a full course of the recommended first-line oral combination therapy: artesunate plus amodiaquine or artemether plus lumefantrine.

Hb levels were assessed by using a HemoCue (Ängelholm, Sweden) Hb201 plus photometer at hospital admission, at discharge, and at follow-up visits on days 7, 14, 21, and 28. To ensure proper functioning of the photometer, high and low liquid controls were tested weekly at each site. No additional biologic or serum markers were investigated.

Of 350 patients treated with intravenous artesunate, 61.4% were children 2–59 months of age. Four (1.1%) patients were referred to another health facility and six (1.7%) died during the treatment period. A total of 270 (77.1%) patients made all four scheduled follow-up visits and successfully completed the full clinical assessment. A total of 41 (11.7%) patients made only one follow-up visit, either at day 21 or at day 28, and 29 (8.3%) did not make any visit.

Of the 270 patients seen at all planned follow-up visits, complete series of Hb measurements (admission, discharge, and four follow-up visits) were available for 201 (57.4%) patients. The proportion of severe anemia cases at admission was 5.5% (11 patients). Among 201 patients with full follow-up, a delayed decrease in the Hb level (2–5 g/dL) was observed in 23 patients (11.4%) between day 7 and day 21 (95% confidence interval = 3.1–36.2%) treated with injectable artesunate. The wide confidence interval highlights the uncertainty of this estimate.

Of these 23 patients, 5 showed a decrease in Hb levels below 5 g/dL on least one of the follow-up visits (Table 1). This decrease was observed eight days after the first dose of artesunate for 1 patient, 15 days after the first dose for 2 patients, and 16 days after the first dose for 1 patient. All four patients received a blood transfusion. A decrease was observed 23 days after the first dose for 1 patient. All five patients had received three doses of artesunate except for one of the patients with anemia on day 15, who had received 5 doses. All patients had completed the parenteral treatment with a full course of artesunate plus amodiaquine. For those patients, Hb levels increased by the day 28 clinical assessment.

We observed in our patients a pattern similar to that reported^4^ with respect to the time of occurrence of anemia (median minimum Hb level at approximately 15 days (Figure 1). The Hb pattern of patient 904 resembled the natural Hb course associated with malaria; the Hb level decreased during the first week post-treatment (early onset). Four patients showed a delayed decrease in the Hb level during the second or third week post-treatment with artesunate. Three patients showed a tendency to normalization of the Hb level by day 28. For patient 913, the Hb level normalized after day 28. Two of the three patients for whom parasitemia results at admission were available were hyperparasitemic, a condition reported to be a potential prognostic factor in a high proportion of patients with delayed hemolytic anemia.^4^ No deaths was observed, and all patients fully recovered at the end of the follow-up period.

**Figure 1.**
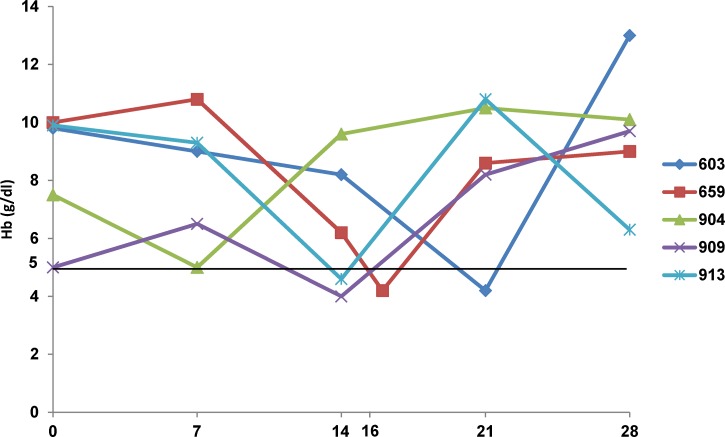
Hb time course of persons with severe anemia at follow-up visits, Democratic Republic of the Congo.

A limitation of this study was that we had no follow-up information for 29 patients because the follow-up was only expanded from 7 to 28 days after the start of the study and no resources were available for home visits. We could therefore not ascertain whether there were severe hemolytic events or deaths in this group. Half of these patients came from one site near the border with Angola, and some of these patients might have crossed the border. However, the site with the largest number of patients enrolled (n = 80) had a follow-up rate of 100%, and no cases of severe delayed anemia or deaths were reported. In addition, three other sites that had 104 patients enrolled had follow-up rates of 95% and no deaths.

Another limitation of the study was that no control group was available and no markers of hemolysis were investigated beyond the Hb level. Thus, it is not possible to associate the delayed decrease in the Hb level observed during the follow-up period with artesunate treatment or disease-specific pathologic processes. In addition, the lack of a control group did not enable measuring the contribution of potential confounders, such as soil-transmitted helminth infections, or reinfections with *P. falciparum* within the 28-day follow-up period.

In a recent study conducted in Gabon and Ghana, delayed hemolysis occurred in 7% of children treated with injectable artesunate,^5^ which is consistent with our findings. Given that artemisinin derivatives could increase the cumulative total dose of artemisinins, thus contributing to an increment in the risk of delayed hemolysis, the risk versus benefit of using alternative antimalarial drugs should be considered. Unfortunately, data for hematologic follow-up of patients after antimalarial treatment are still scarce, including for quinine treatment. A study of imported malaria cases in the United Kingdom reported a decrease in Hb levels in 61% of cases 5–21 days after treatment with quinine.^6^

The proportion of patients with severe anemia in our study groups was below 1% for the whole duration of the follow-up period. In all cases, delayed anemia was clinically manageable with appropriate and prompt care. The outcomes of this study supports recent WHO recommendations^7^ for continued use of injectable artesunate as a life-saving treatment and that healthcare professionals should be made aware of the potential for delayed hemolytic anemia for up to one month post-treatment and encouraged to actively monitor patients during this period. Our data support the need for strengthening pharmacovigilance systems as recommended by a meeting convened by Medicines for Malaria Venture that involved malaria experts working in the field of severe malaria.^8^

## Figures and Tables

**Table 1:** Summary data of patients with life-threatening anemia during follow-up visits, Democratic Republic of Congo*

Case no.	Age	AP (parasites/μL	PCT (hours)	Hb level at admission (g/dL)	Minimum Hb level (g/dL)	Day of lowest Hb decrease	Hb level at day 28 (g/dL)
603	3 years	435,815	NA	9.8	4.2	23	13.0
659	30 months	199,299	NA	10	4.2†‡	16	9.0
904	26 months	NA	24	7.5	5.0†	8	10.5
909	6 months	53,293	23.6	5.0†	4.0†	15	9.7
913	8 months	NA	24	9.9	4.6†	15	6.3

* AP = admission parasitemia; PCT = parasite clearance time; HB = hemoglobin; NA = not available.

† These patients received a blood transfusion.

‡ This patient had an abscess at the injection site that was treated on day 16 and the Hb level then increased.
